# Reproductive Longevity and Aging: Geroscience Approaches to Maintain Long-Term Ovarian Fitness

**DOI:** 10.1093/gerona/glaa204

**Published:** 2020-08-18

**Authors:** Natalia Llarena, Christopher Hine

**Affiliations:** 1 Department of Cardiovascular and Metabolic Sciences, Cleveland Clinic Lerner Research Institute, Ohio; 2 Reproductive Endocrinology and Infertility, Cleveland Clinic Women’s Health Institute, Ohio

**Keywords:** Delayed childbearing, mTOR, Ovarian aging, Ovarian reserve, Primordial follicles

## Abstract

Increases in delayed childbearing worldwide have elicited the need for a better understanding of the biological underpinnings and implications of age-related infertility. In women 35 years and older the incidences of infertility, aneuploidy, and birth defects dramatically increase. These outcomes are a result of age-related declines in both ovarian reserve and oocyte quality. In addition to waning reproductive function, the decline in estrogen secretion at menopause contributes to multisystem aging and the initiation of frailty. Both reproductive and hormonal ovarian function are limited by the primordial follicle pool, which is established in utero and declines irreversibly until menopause. Because ovarian function is dependent on the primordial follicle pool, an understanding of the mechanisms that regulate follicular growth and maintenance of the primordial follicle pool is critical for the development of interventions to prolong the reproductive life span. Multiple pathways related to aging and nutrient-sensing converge in the mammalian ovary to regulate quiescence or activation of primordial follicles. The PI3K/PTEN/AKT/FOXO3 and associated TSC/mTOR pathways are central to the regulation of the primordial follicle pool; however, aging-associated systems such as the insulin-like growth factor-1/growth hormone pathway, and transsulfuration/hydrogen sulfide pathways may also play a role. Additionally, sirtuins aid in maintaining developmental metabolic competence and chromosomal integrity of the oocyte. Here we review the pathways that regulate ovarian reserve and oocyte quality, and discuss geroscience interventions that leverage our understanding of these pathways to promote reproductive longevity.

Worldwide trends toward delayed childbearing have brought to the forefront the impact of age-related infertility. The average age of first birth has increased from 21 in 1970 to 26.9 in 2018, and the proportion of women having their first child after age 35 increased nearly 10-fold since 1970 (1). After age 35, rates of infertility, aneuploidy, and birth defects increase dramatically. Although in vitro fertilization (IVF) can successfully address infertility in younger women, it cannot reverse the effects of age, particularly in women over 40 (2). Age is the driving factor affecting IVF outcomes (3). Live birth rates after IVF decrease from 46.8% in women under 35 to 3.1% after the age of 42, a trend that parallels spontaneous conception rates (4). Thus, research into mechanisms that regulate and preserve reproductive health is of great importance.

In addition to diminished reproductive function, the withdrawal of estrogen during menopause due to ovarian failure has systemic consequences, including cardiovascular disease, skeletal fragility, and genitourinary and vasomotor symptoms (5,6). The average age of menopause in the United States is 51; therefore, the majority of women are likely to spend at least 30 years of life in the postmenopausal period. Women may also undergo menopause prematurely due to gonadotoxic chemotherapy (7) or genetic disorders such as Turner’s syndrome (8) and Fragile X (9). Premature ovarian insufficiency (POI) increases the risk for both increased total mortality as well as mortality related to ischemic heart disease (10–12).

Both reproductive and hormonal ovarian function are limited by the primordial follicle pool (PFP). The PFP, which constitutes the ovarian reserve, is established in utero and irreversibly declines until menopause. Because ovarian function is dependent on the PFP, an understanding of the mechanisms that regulate follicular growth and maintenance of the PFP is critical for the development of interventions that prolong reproductive life span and endocrine homeostasis. The following review addresses mechanisms regulating the PFP and oocyte quality (Mechanisms of Ovarian Aging section and Supplementary Table 1), while providing insight into geroscience interventions designed to leverage these mechanisms (Leveraging Geroscience Approaches to Maintain Long-Term Ovarian Fitness and Promote Reproductive Longevity section and Supplementary Table 2).

## Mechanisms of Ovarian Aging

### Follicular Maturation

Exit of follicles from the PFP is the critical event that depletes ovarian reserve. A primordial follicle can (a) remain in a dormant state, (b) be activated to undergo follicular maturation, or (c) undergo atresia from its quiescent state. Although the fate of most follicles is atresia, during each cycle, a cohort of follicles is “recruited” to undergo follicular maturation. Once a cohort of follicles is selected for maturation, it becomes responsive to follicle-stimulating hormone, estrogen, and luteinizing hormone. A dominant follicle emerges and undergoes ovulation, while the remaining cohort undergoes atresia. This process repeats throughout female human reproductive life span, during which approximately 400 follicles will ovulate.

The concept that the PFP is fixed from birth is generally accepted (13). However, Tilly et al identified cells in the bone marrow of adult female mice that, when transplanted, produced oocytes within immature follicles (14–16). Additionally, oogonial “precursor cells” expressing stem cell markers have been obtained from the ovarian cortex in postmenopausal women, suggesting the possibility of neo-oogenesis during adulthood (17,18). Regardless of whether postnatal ovaries are capable of oocyte production, it is clear that the ovaries senesce long before other organ systems, and that a thorough understanding of folliculogenesis and the mechanisms that maintain the PFP is critical for developing interventions to prolong reproductive life span and ovarian health.

### Regulation of the Ovarian Reserve by Mammalian Target of Rapamycin and Transsulfuration/Hydrogen Sulfide

Once a follicle has been activated out of the primordial pool and follicular maturation has been initiated, the process is irreversible, resulting either in ovulation or atresia (19). Increased activation of primordial follicles is partially driven by augmented phosphatidylinositol 3 kinase (PI3K) and mammalian target of rapamycin (mTOR) activity (20). In the oocyte, follicular activation occurs when PI3K converts PIP2 into PIP3 (21). PIP3 stimulates protein kinase B (AKT) activation and nuclear translocation, which reduces the transcriptional activity of forkhead box O3 (FOXO3) by phosphorylation (21). While suppressors of follicular activation, such as FOXO3a, tuberous sclerosis complex 1 (TSC1), and tuberin (TSC2), are deactivated by phosphorylation, the phosphorylation of Akt, mTOR, and ribosomal protein S6 (rpS6), a downstream actor of mTOR signaling, results in follicular activation and growth. Ovaries obtained from mice after chemotherapeutic stress demonstrated phosphorylation of Akt, mTOR, and rpS6, as well FOXO3a (19).

Interestingly, primordial follicles begin transcribing genes for follicular growth as early as postnatal day 1 (22); in essence they are “preloaded” for maturation (22). This suggests that prevention of follicular maturation requires translational suppression of these transcripts (21), leading to the hypothesis that mTOR, as a master regulator of protein translational and cellular growth (23), plays a strong role in this process. Supporting this theory is that oocyte-specific deletion of the mTOR inhibitory factor TSC1 in mice stimulated mTOR complex 1 (C1) and activated the entire PFP at sexual maturity, leading to POI (20). Additionally, the Fragile X mental retardation protein (FMRP), encoded by the *FMR1* gene, is highly expressed in oocytes (24) and the *FMR1* premutation is a common genetic etiology of POI (24). Fmr1 KO mice expressed higher levels of mTOR (25), and utilization of the mTOR inhibitor rapamycin alleviated POI, increased the PFP, and extended age of last litter (24). Likewise, utilization of rapamycin enhanced reproductive longevity in wild-type mice (26). These studies suggest that activation of the mTOR pathway is associated with irreversible activation of primordial follicles, while mTOR inhibitors serve as pharmacological leads to reduce depletion of the ovarian reserve in women at risk of premature menopause due to genetic or genotoxic factors.

The transsulfuration pathway and its related hydrogen sulfide (H_2_S) production/metabolism are antiaging targets that have recently been studied for promoting longevity, health span, and fitness in multiple organ systems and organisms (27–30). Emerging data suggest that these pathways interface with mTOR and play a role in ovarian function. Enhanced enzymatically produced endogenous H_2_S plays multiple beneficial signaling, antioxidant, and metabolic roles across evolutionary boundaries for life span and/or health-span extension (27). Hydrogen sulfide is largely produced via the transsulfuration pathway enzymes cystathionine β-synthase (CBS) and cystathionine γ-lyase (CGL), along with the non-transsulfuration pathway enzyme 3-mercaptopyruvate transferase (3MST) (31,32). Hydrogen sulfide production and signaling decline with age (33,34). Constitutive activation of mTOR in mouse liver reduces CGL expression and H_2_S production (28). Additionally, long-lived mice lacking ribosomal protein S6 kinase (S6K1), a crucial downstream factor in mTOR signaling, have increased hepatic CGL expression (35). However, little is known about the role of H_2_S and/or mTOR-regulated transsulfuration pathway regulation in the ovary. Expression of CBS and CGL mRNA is detected in granulosa, cumulus, and oocyte cells (36). Female CGL knockout mice are fertile, possibly in a strain-specific manner; however, reproductive outcomes have not been evaluated at advanced ages (37,38). Cystathionine β-synthase knockout is typically lethal in mice before the age of 5 weeks; however, surviving females are infertile (39). Endogenous H_2_S production participates in the preovulatory cascade and follicular rupture during ovulation in mice (36). Outside of the ovary, CBS and CGL are abundantly distributed in the fallopian tube epithelium and inhibition of their H_2_S production causes embryo retention and delays oviductal embryo transport (40). Interestingly, increased cardioprotective CGL-derived H_2_S production may be a downstream target of estrogen signaling (41), suggesting a potential mechanism of action for postmenopausal increases in cardiovascular disease in women. Further research is needed to elucidate how aging-related losses in H_2_S production and signaling play in ovarian and reproductive decline and vice versa.

### Growth Hormone and the Insulin-Like Growth Factor-1 Pathway

Although growth hormone (GH)- and GH receptor (GHR)-deficient mice have extended life span, health span, and increased ovarian reserve, data about the impact of GH on ovarian function are inconsistent (42). Growth hormone receptor knockout (GHRKO) mice have a larger PFP and smaller number of growing follicles than wild-type mice, suggesting a role for GH in follicular recruitment; however, GHRKO mice also show an increased proportion of atretic follicles (43). Consistent with these findings, treatment of GHRKO mice with insulin-like growth factor-1 (IGF-1) decreases the PFP, increases the proportion of healthy antral follicles, and decreases follicular atresia (43). Similarly, transgenic mice that overexpress GH demonstrate decreased ovarian reserve (44). The effects of GH and IGF-1 appear to be mediated by FOXO3a, as GH-deficient mice have lower circulating levels of phosphorylated FOXO3a (44). Phosphorylation of FOXO3a releases the brake on primordial follicle activation. Despite these observations, there are circumstances in which GH and IGF-1 appear to be protective for the ovarian reserve. For instance, GH, which induces IGF-1 transcription and translation in the ovaries, has been shown to be protective in a rat model of radiation-induced ovarian injury (45). Cotreatment with GH during radiation resulted in increased anti-Müllerian hormone (AMH) levels and primordial follicle counts compared to controls (45). Additionally, the selective estrogen receptor modulator tamoxifen increases AMH and IGF-1 expression in the ovaries and protects against radiation-induced ovarian injury (46). Further data are needed to clarify these paradoxical findings with regard to natural aging and genotoxic stress-induced premature aging.

### The Role of AMH in Maintaining the Ovarian Reserve

Anti-Müllerian hormone serves as another gatekeeper of the PFP. Also known as Müllerian-inhibiting substance, AMH is a member of the transforming growth factor-beta family. In adult women, AMH is produced by granulosa cells of small growing follicles, and regulates recruitment and maturation of follicles from the PFP (47). Anti-Müllerian hormone prevents PFP depletion by reducing phosphorylation and maintaining activation of FOXO3a, a downstream component of the PI3K/PTEN/AKT pathway that helps to maintain the dormancy of primordial follicles (21,48) and prevents follicular recruitment (49–51). Consistent with this hypothesis is the observation that *Amh* knockout mice are initially fertile, but undergo premature depletion of the follicular pool, demonstrating a loss of the PFP by 13 months of age (52). Likewise, AMH serves as a clinical marker of ovarian reserve and response to ovarian stimulation for IVF (47).

Due to the role of AMH in preventing activation of primordial follicles, providing exogenous AMH as an intervention to reduce chemotherapy-induced gonadal injury and premature aging has been proposed. In mice, exogenous AMH reduced primordial follicle loss in the setting of exposure to carboplatin and doxorubicin (53). Moreover, AMH treatment induced a complete, but reversible, shutdown of folliculogenesis, supporting the hypothesis that the hormone serves as a gatekeeper of follicular activation out of the PFP (53).

### The Role of Mitochondria and Reactive Oxygen Species in Ovarian Aging

The mature mammalian oocyte is highly enriched in mitochondria and contains a larger mitochondrial DNA (mtDNA) copy number than any other cell type (54). Interestingly, mtDNA copy number is increased dramatically during oocyte maturation, from approximately 100 in primordial germ cells to over 100 000 in mature oocytes (55,56). This observation has led to the “bottleneck hypothesis,” in which a small number of mtDNA copies contribute to the mitochondria population in a new organism. This mechanism is hypothesized to promote mitochondrial integrity across generations and minimize the population of organisms with abnormal mtDNA (56). Thus, maintaining mtDNA integrity and function is of utmost importance for ovarian and reproductive health.

However, mtDNA point mutations and rearrangements amassed during aging, either through reactive oxygen species (ROS)-induced damage or improper mtDNA replication and repair, ultimately lead to compromised cellular energy production and further ROS production (57,58). Reactive oxygen species generation is associated with germ cell apoptosis and poor oocyte quality (59,60). Increased levels of ROS in human follicular fluid are predictive of impaired embryo development and embryo arrest in IVF cycles (61). In a mouse model of insulin resistance, germinal vesicles and metaphase II oocytes were found to have increased ROS, impaired mitochondrial function, and demonstrated a high rate of apoptosis, with surviving oocytes of poor quality with abnormal meiotic spindles and misaligned chromosomes (60).

In both mice and humans, a proof-reading deficiency of PolgA, a nuclear-encoded catalytic subunit of mtDNA polymerase, results in accumulation of mtDNA mutations (62–65). Homozygous knockin mice expressing a proof-reading-deficient version of PolgA accumulate 3–5 times more mtDNA mutations than wild-type mice and demonstrate reduced fertility (63). The effect of deficient PolgA on ovarian function is profound, as female knockin mice are not able to become pregnant after 20 weeks of age (63). Similarly, a human study of 7 families with PolgA mutations demonstrated that most women with PolgA deficiencies undergo menopause before the age of 35 (64). It is likely the accumulation of mtDNA mutations through ROS-induced mtDNA damage or impaired mtDNA repair is part of a complex series of mechanisms that contribute to ovarian aging.

Dysfunctional mitochondrial fission and fusion mechanisms represent additional drivers in ovarian aging. In mammals, mitochondrial fusion requires 3 GTPases: mitofusin (MFN1), mitofusin 2 (MFN2), and optic atrophy gene 1 (OPA1) (66). Fission requires recruitment of dynamin-related protein 1 to the outer mitochondrial membrane. After it is recruited, dynamin-related protein 1 constricts mitochondrial tubules to allow for membrane fission (66). Oocyte-specific deletions of these proteins impaired oocyte function and follicular development. Deletion of both *Mfn1* and *Mfn2* from mouse oocytes results in poor oocyte maturation, infertility, increased oocyte apoptosis, and accelerated follicular depletion (67,68). Additionally, knockout of *Drp1* in the oocyte demonstrates that fission is also necessary for follicular maturation and ovulation (69). Together, these data demonstrate the critical role of mitochondrial function in maintaining ovarian reserve, oocyte quality, and normal follicular development.

### The Role of Nicotinamide Adenine Dinucleotide-Dependent Deactylases/Sirtuins in Ovarian Aging

Sirtuins (SIRT) are a family of nicotinamide adenine dinucleotide-dependent deactylases that control cellular metabolism, proliferation, and genome stability (70). They are central in regulating aging across evolutionary boundaries (71). Several studies have demonstrated a role for sirtuins in ovarian aging (70,72–74). Mainly, caloric restriction (CR) that confers reproductive longevity results in elevated SIRT1 and SIRT6 levels in ovaries (72–74), while transgenic mice overexpressing ovarian SIRT1 demonstrate suppressed levels of mTOR and longer ovarian life span (72). Conversely, SIRT1 and SIRT3 deficiencies accelerate loss of the PFP and increased mTOR signaling (75). Together, these data suggest that decreased sirtuin expression is associated with ovarian aging, in part through the loss of mTOR inhibition.

There also appears a role for sirtuins to regulate quality and developmental competence of oocytes. Chromatin compaction, which increases from early to medium antral follicle stage, is an indicator of widespread transcriptional silencing, oocyte differentiation, and a marker of developmental competence in multiple species that is partially driven by increased SIRT1 and SIRT6 activity (75–77). During chromatin compaction H3K9 deacetylation is required; however, aging-related decreases in ovarian SIRT1 expression negatively influence chromatin compaction and lead to impaired oocyte development (75). The administration of NAM, a “noncompetitive pan-sirtuin inhibitor,” prevents entry of mouse oocytes into meiosis I and results in meiosis II arrest (75,78). Similarly, the administration of the SIRT1 inhibitor EX527 to mice increases ROS and abnormal metaphase II plates in the oocyte (75). Whereas SIRT2 knockdown impairs spindle organization and chromosome alignment, SIRT3 overexpression reduces spindle defects and chromosome misalignment in mouse oocytes (75,79). These data demonstrate a consistent role for sirtuins in the regulation of meiotic spindle assembly, through control of oxidative stress, oocyte development, and chromosome segregation during meiosis.

## Leveraging Geroscience Approaches to Maintain Long-Term Ovarian Fitness and Promote Reproductive Longevity

Manipulating the rate of aging and delaying the onset of aging-related diseases have been the makeup of medical, scientific, and pseudoscientific pursuits throughout history. However, it is not until relatively recently, in the later part of the 20th and early 21st centuries, that the molecular targets and geroscience approaches needed to make this a reality have been elucidated. While these interventions have primarily focused on 3 primary aims; life span, metabolic fitness, and stress resistance, they have often neglected female reproductive health. Below and in Supplementary Table 2, we address the current state and application of common geroscience approaches as they relate to reproduction and ovarian health.

### Dietary Restriction

Dietary restriction (DR) increases life span across multiple model organisms and confers protection against cancer, diabetes, atherosclerosis, autoimmune conditions, and neurodegenerative disease (80). Dietary restriction encompasses a number of dietary interventions, including CR, sulfur amino acid restriction, fasting, and protein restriction (27). Principally, CR increases ovarian reserve, improves oocyte quality, and prolongs the reproductive life span in mammals (81,82). However, during the period of CR, estrous cycles are disrupted, fertility is poor, and survival outcomes for offspring are compromised in mice (82). Similarly, humans with insufficient caloric intake experience infertility due to anovulation (83). Nevertheless, diminished fertility during periods of stress, such as that induced by CR, engages defense mechanisms that preserve reproductive potential once the stressor is removed and/or refeeding begins (83).

Although most rodents do not have menses or undergo a true menopause, they do experience declines in ovarian reserve and oocyte quality with age. In mice, declining fertility begins after 10 months of age, and female mice are typically infertile by 15 months of age (82). Moderate (40%) CR increases their reproductive life span and improves oocyte quality (82,84). In a mouse model, 10% CR was initiated at 14 weeks of age, gradually increased to 40% at 16 weeks, and maintained until 15.5 months of age, when mice resumed an ad libitum diet. Whereas ad libitum-fed controls became infertile at 15.5 months, mice who underwent CR remained fertile until 23 months. Additionally, offspring survival rates were improved in the CR cohort. Only 22% of offspring born between 10 and 23 months of age in the ad libitum-fed group survived, whereas over 73% of pups born to the CR cohort survived (82,85). The improvements in long-term reproductive outcomes with CR can partially be explained by improvements in oocyte quality, as dietary-restricted mice do not demonstrate age-related increases in oocyte aneuploidy, meiotic spindle abnormalities, chromosomal misalignment at the metaphase plate, or impaired mitochondrial function (84). These data suggest CR improved both ovarian reserve and oocyte quality.

Ratios of macronutrients independent of caloric intake also have marked effects on rodent reproductive outcomes (86). A high-fat diet results in a large number of follicles, but few corpora lutea, suggesting anovulation and infertility. Conversely, the highest number of ovulatory cycles occurred under a low protein to carbohydrate ratio of 1:8, with maximum longevity obtained with a protein to carbohydrate ratio of 1:11 (86). In the fly, CR with methionine supplementation increases life span without compromising fertility, suggesting that the mechanisms that control life span and fecundity are distinct and reallocating nutrients may allow for survival benefits while retaining fertility (87). Interestingly, flies fed a diet designed to match the amino acid proportion in the *Drosophila melanogaster* exome enhanced growth and reproduction (88).

Several mechanisms are proposed to account for the benefits of DR, including modulation of mTOR, GH/IGF-1, and/or sirtuin activity and related signaling. A 10-week period of CR in mice increased the number of primordial follicles and decreased ovarian p70S6k signaling, suggesting that CR may prolong the reproductive life span by inhibiting mTOR signaling in the ovary (81). The effects of CR on mTOR may be mediated in part by SIRT1. SIRT1, which is induced by CR, negatively regulates mTOR signaling in both human and mouse cells in vitro, highlighting the interface of these pathways (89). Additionally, CR reduces circulating levels of IGF-1, and exposure of cells to insulin or IGF-1 decreases the expression of SIRT1 (90). GH/GHR deficient mice have extended life span, health span, and reproductive potential (42,91). Although the effects of DR on the transsulfuration/H_2_S pathway in the ovary have not been established, mTOR (28) and GH/IGF-1 (29) serve as negative regulators of H_2_S production in other tissues, allowing for the possibility of similar mechanisms in the ovary.

Whether the reproductive longevity benefits of DR extend to humans remains to be seen. However, 2 randomized trials demonstrate the safety and feasibility of CR in small cohorts, and show that it is effective at decreasing metabolic, hormonal, and inflammatory risk factors for cardiovascular disease, diabetes, and cancer in humans (92–95). At a minimum, the studies of DR in model organisms facilitate a molecular understanding of pathways conferring reproductive longevity and long-term ovarian health so as to be further leveraged for targeted dietary and nondietary clinical interventions, including those described below.

### Rapamycin

In humans, the mTORC1 inhibitor rapamycin is FDA-approved as an immunosuppressant in transplant recipients and for treatment of lymphangioleiomyomatosis; additionally, mTOR inhibitors are increasingly being used for cancer treatment (96). Concerns about rapamycin’s side effects, which include immunosuppression and glucose intolerance, have limited its human trials for promoting longevity (97). In mice, rapamycin extends life span and health span, and induces improvements in cognitive function, activity levels, and cardiac function even when initiated late in life (98–101). Rodent studies demonstrate the antiaging effects of rapamycin observed in other organ systems also extend to the ovary. Improvements in reproductive function after rapamycin treatment are evident in studies of physiologic murine aging, as well as in models of chemotherapy-induced POI. A 2-week course of rapamycin in healthy mice improved primordial follicle count, oocyte morphology, and mitochondrial activity (26). In mating studies, after 12 months of age, when the control mice began to experience age-related infertility, the rapamycin-treated mice retained fertility and continued to have pups. Improved reproductive longevity appeared when rapamycin was either initiated at 8 weeks or 8 months (26). As in studies of CR, estrous cycles are irregular and fertility is poor during the period of rapamycin treatment, but recover within 2 months of rapamycin discontinuation (26). Rapamycin-treated animals demonstrated increased ovarian expression of SIRT1 and SIRT6, as well as decreased abundance of phosphorylated mTOR and p70S6k (102). Additionally, rapamycin and its analogue everolimus reduced loss of primordial follicles and maintained fertility in mating studies in mice that underwent treatment with cyclophosphamide, suggesting the potential for fertility preservation in humans undergoing chemotherapy (103–105).

### Metformin

Metformin is an antidiabetic biguanide that improves insulin sensitivity. It acts on complex I of the mitochondrial respiratory chain and inhibits mTORC1 indirectly by activating TSC2 (106). Metformin has been shown to increase life span and health span in both lower organisms and rodents (107–109); however, some data suggest that these outcomes are strain and sex-specific (110,111).

Use of metformin in reproductive medicine is limited to women with polycystic ovarian syndrome. It safely and effectively improves ovulation rates in the polycystic ovarian syndrome population, and increasing evidence demonstrates safety during the first trimester of pregnancy (112). In mice, 6-month courses of metformin increase ovarian reserve, potentially by inducing SIRT1 expression and reducing oxidative stress (113); however, other studies have failed to demonstrate a benefit of metformin on reproductive longevity (114). Further research is needed to evaluate the impact of metformin on ovarian aging.

### Resveratrol

Resveratrol is a polyphenolic compound initially identified as a SIRT1 activator in yeast (115,116). In aging mice, resveratrol diminishes age-related deterioration by reducing inflammatory markers and cataract formation while improving aortic elasticity, motor coordination, and bone density; however, it does not increase overall longevity (117,118). In humans, randomized trials evaluating the impact of resveratrol have been mixed. A small randomized trial of 20 adults showed that a 6-week course of resveratrol supplementation reduced plasma concentrations of the inflammatory markers C-reactive protein and tumor necrosis factor (119); however, another phase 2 trial of oral resveratrol in multiple myeloma patients was discontinued due to a high rate of renal failure and adverse effects (120). More recently, randomized trials have demonstrated a benefit of resveratrol on lipid profiles in patients with risk factors for cardiac disease (121,122).

With regard to reproductive outcomes, a 12-month course of resveratrol in mice increased primordial follicle counts, litter size, and oocyte quality at advanced ages (116,123). Additionally, a specific SIRT1 activator SRT1720 administered to mice suppressed the activation of primordial follicles and increased the ovarian reserve by activating SIRT1 and inhibiting mTOR signaling (124). A recent human trial randomized 61 women with polycystic ovarian syndrome undergoing IVF to a 40-day course of resveratrol versus placebo. Resveratrol treatment resulted in improved oocyte and embryo morphology; however, this did not result in a change in oocyte fertilization rate or pregnancy rate (125). Given the favorable safety profile of resveratrol, it is reasonable to pursue further randomized trials in human fertility patients to further evaluate whether resveratrol has the capacity to improve reproductive function and mitigate ovarian aging.

### Melatonin

Melatonin is an indoleamine neurotransmitter secreted primarily by the pineal gland in a pulsatile fashion. Its primary role is in the regulation of sleep–wake cycles. Melatonin is also synthesized by the oocyte, granulosa cells, cumulus cells, and the placenta, where it is thought to facilitate oocyte maturation, mitigate oxidative stress, and optimize placental function by preventing apoptosis (126). Consistent with its role in regulating circadian rhythms, melatonin also contributes to seasonal reproduction in mammals. The duration of elevated melatonin and the direction of change in melatonin levels both contribute to ovarian function in hamsters and sheep (127,128). The pulsatile secretion of melatonin is believed to be important for maintaining a functioning pituitary–ovarian axis in humans, and high doses of exogenous melatonin have a suppressive effect on ovulation in women (129).

Peak melatonin levels decrease with age, and melatonin supplementation may have a number of benefits, including increased life span, improved immune function, and reproductive longevity (130). In mice, transplanting the pineal gland from a young mouse into an aging animal improved immune function and increased life span (131). Additionally, multiple melatonin supplementation studies in rodents demonstrated improvements in markers of ovarian health, including increased litter size, increased number of primordial follicles, and improved oocyte competence (132–135). Mice with melatonin supplementation additionally showed improved mitochondrial antioxidant function, reduced ovarian mitochondrial ROS generation, and increased SIRT1 and SIRT3 activity in granulosa cells (132,135). Additionally, mice exposed to genotoxic cisplatin that received pretreatment with melatonin had significantly less primordial follicle loss and decreased granulosa cell apoptosis; these effects were mediated by a decrease in phosphorylated FOXO3 (136).

A study of women undergoing IVF demonstrated that melatonin supplementation protected oocytes from oxidative damage in vitro and improved fertilization rates (137). Similarly, a recent prospective study of 40 women with unexplained infertility evaluated the impact of melatonin supplementation (3 mg or 6 mg daily) for 40 days on IVF outcomes and found an increase in intrafollicular antioxidants, as well as improved fertilization rates from 47% to 67% (138). There was no change in live birth rate; however, the study was not powered to evaluate live birth rate as an outcome (138). Some data also suggest that melatonin improves hormonal function in peri- and postmenopausal women. A randomized trial of 79 women aged 42–62 evaluated the impact of a 6-month course of melatonin (3 mg nightly) on follicle-stimulating hormone levels, which is a biomarker of menopause that increase with age. The authors reported a significant reduction in follicle-stimulating hormone levels after melatonin treatment and a resumption of menstrual cycles in several postmenopausal women (139). Together, these data suggest that melatonin may have a role in mitigating ovarian aging and improving oocyte quality. The favorable safety profile of melatonin make it an appealing target for future research.

## Conclusions

**Figure 1.**
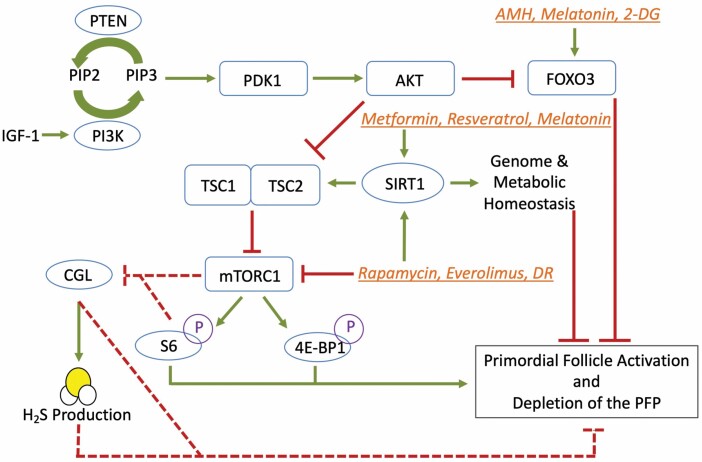
Aging-related pathways and their role in ovarian aging and regulation of the primordial follicle pool. Multiple pathways, including PI3K/PTEN/AKT/FOXO3, TSC/mTOR, growth hormone (GH)/IGF-1, H_2_S, and sirtuins, converge in the mammalian ovary to regulate the quiescence and activation of primordial follicles. Behavioral and pharmacologic interventions (orange italic font) to prevent depletion of the ovarian reserve target distinct points along these pathways. Dotted lines represent areas of emerging research, whereas as solid lines represent established pathways within the mammalian ovary. Abbreviations used are as follows: insulin-like growth factor-1 (IGF-1); phosphatidylinositol bisphosphate (PIP2); phosphatidylinositol triphosphate (PIP3); phosphoinositide-dependent kinase-1 (PDK1); protein kinase B (AKT); forkhead box O3 (FOXO3); phosphatidylinositol 3 kinase (PI3K); anti-Müllerian hormone (AMH); sirtuin 1 (SIRT1); tuberous sclerosis complex 1 (TSC1); tuberin (TSC2); mammalian target of rapamycin (mTOR) complex 1 (mTORC1); dietary restriction (DR); 2-deoxyglucose (2-DG); cystathionine γ-lyase (CGL); ribosomal protein S6 (S6); eukaryotic initiation factor 4E-binding protein 1 (4E-BP1); H_2_S (hydrogen sulfide); primordial follicle pool (PFP).

Multiple pathways, many of them nutrient-sensing, converge in the mammalian ovary to regulate the quiescence and activation of primordial follicles. The PI3K/PTEN/AKT/FOXO3 and TSC/mTOR pathways appear to be central to the regulation of the PFP; however, GH/IGF-1 and H_2_S may also play a role (Figure 1). A delicate balance of primordial follicle activators and suppressors must be maintained in order to allow for continued ovulation while preventing rapid depletion of the ovarian reserve. The behavioral and pharmacologic interventions that prevent primordial follicle activation, including DR and rapamycin, cause infertility for the duration of the intervention. In order for these interventions to be useful clinically, the resulting period of infertility must be reversible, and the treatments must confer long-term benefits after a relatively short duration of use. Perhaps the best candidates for pharmacologic inhibitors of primordial follicle activation are women undergoing gonadotoxic chemotherapy, or those known to be at high risk for POI, such as women carrying the FMR1 premutation. Initial data from human trials suggest that short courses of pharmacologic SIRT1 inducers such as resveratrol and melatonin may improve oocyte morphology and fertilization rates in women undergoing IVF; however, more data are needed to confirm these findings. Ultimately, as women age, the risks of infertility, aneuploidy, and miscarriage increase due to a decline in both ovarian reserve and oocyte quality. Enhanced understanding of the pathways that maintain the ovarian reserve presented in this review and the geroscience interventions that command them will promote reproductive longevity and extend ovarian-related endocrine homeostasis into later life.

## Funding

This work was supported by the National Institutes of Health (grant numbers R00 AG050777 and R01 HL148352 to C.H.).

## Conflict of Interest

None declared.

## Supplementary Material

glaa204_suppl_Supplementary_Material
