# Supplementary material for: Reproductive Longevity and Aging: Geroscience Approaches to Maintain Long-Term Ovarian Fitness
Source: J Gerontol A Biol Sci Med Sci. 2020 Aug 18;76(9):1551–60. doi: 10.1093/gerona/glaa204 (PMC8361335; doi:10.1093/gerona/glaa204)
Supplement: glaa204_suppl_Supplementary_Material [file glaa204_suppl_supplementary_material.pdf]

eTable 1. Mouse models of premature ovarian insufficiency

Model (alphabetical)	Organism	Phenotype	Citation
AMH knockout	Mouse	Significantly smaller number of primordial follicles at 4 months and 13 months	Durlinger, A. L. L. Control of Primordial Follicle Recruitment by Anti-Mullerian Hormone in the Mouse Ovary. *Endocrinology* (1999). doi:10.1210/en.140.12.5789
CBS knockout	Mouse	Severe growth restriction; typically die within 5 weeks; surviving female are infertile.	Watanabe, M. *et al.* Mice deficient in cystathionine β-synthase: Animal models for mild and severe homocyst(e)inemia. *Proc. Natl. Acad. Sci. U. S. A.* (1995). doi:10.1073/pnas.92.5.1585
CLPP knockout	Mouse	Infertility; decreased response to ovarian stimulation and poor embryo development; increased rate of abnormal oocyte meiotic spindles; accelerated depletion of the ovarian reserve (decreased number of primordial and primary follicles at 24 weeks); increased ROS production; impaired mitochondrial dynamics	Seli, E., Wang, T. & Horvath, T. L. Mitochondrial unfolded protein response: a stress response with implications for fertility and reproductive aging. Fertility and Sterility (2019). doi:10.1016/j.fertnstert.2018.11.048
DRP1 deletion (oocyte-specific)	Mouse	Decreased fecundity due to impaired follicular maturation and ovulation; impaired granulosa cell proliferation; abnormal oocyte mitochondrial morphology; oocyte mitochondrial aggregation; impaired intercelluar communication between granulosa cells and oocytes	Udagawa, O. *et al.* Mitochondrial fission factor Drp1 maintains oocyte quality via dynamic rearrangement of multiple organelles. *Curr. Biol.* (2014). doi:10.1016/j.cub.2014.08.060
FMR1 knockout	Mouse	Decreased primordial follicle count at 18 weeks; depletion of primordial follicle pool by 40 weeks; decreased fecundity after 29 weeks	Mok-Lin, E. *et al.* Premature recruitment of oocyte pool and increased mTOR activity in Fmr1 knockout mice and reversal of phenotype with rapamycin. *Sci. Rep.* (2018). doi:10.1038/s41598-017-18598-y
FOXO3a knockout	Mouse	Overactivation of the primordial follicle pool and POI by 2 weeks	Castrillon, D. H., Miao, L., Kollipara, R., Horner, J. W. & DePinho, R. A. Suppression of ovarian follicle activation in mice by the transcription factor Foxo3a. *Science (80-.).* (2003). doi:10.1126/science.1086336
MFN1 deletion (oocyte-specific)	Mouse	Infertility due to follicular arrest at the secondary follicle stage; accelerated follicular depletion (lower number of primordial and primary follicles at 24 weeks); impaired intercellular communication between oocyte	Zhang, M. et al. Mitofusin 1 is required for female fertility and to maintain ovarian follicular reserve. Cell Death Dis. (2019). doi:10.1038/s41419-019-1799-3

		and granulosa cells; impaired mitochondrial function	
MFN2 deletion (oocyte-specific)	Mouse	Poor ovarian response to stimulation; significant decrease in primordial follicle pool at 24 weeks and decreased number of follicles at all stages; increased rate of oocyte meiotic spindle abnormalities; increased follicular apoptosis; lower levels of ATP production by oocyte mitochondria	Zhang, M. *et al.* Mitofusin 2 plays a role in oocyte and follicle development, and is required to maintain ovarian follicular reserve during reproductive aging. *Aging (Albany. NY).* (2019). doi:10.18632/aging.102024
PolgA proof-reading deficient knock-in	Mouse	Three-to-fivefold increase in the number of mtDNA point mutations; global premature aging phenotype starting at 25 weeks, including weight loss, alopecia, kyphosis, osteoporosis, anemia, and cardiomegaly; reduced median lifespan (48 weeks); infertility with no pregnancies after 20 weeks	Trifunovic, A. *et al.* Premature ageing in mice expressing defective mitochondrial DNA polymerase. *Nature* (2004). doi:10.1038/nature02517
PTEN deletion (oocyte-specific)	Mouse	Overactivation of the primordial follicle pool and POI by 12-13 weeks	Reddy, P. *et al.* Oocyte-specific deletion of pten causes premature activation of the primordial follicle pool. *Science (80-.).* (2008). doi:10.1126/science.1152257
TSC1 deletion (oocyte-specific)	Mouse	Overactivation of the primordial follicle pool and POI by 12-13 weeks	Adhikari, D. et al. Tsc/mTORC1 signaling in oocytes governs the quiescence and activation of primordial follicles. Hum. Mol. Genet. (2009). doi:10.1093/hmg/ddp483

eTable 2. Geroscience approaches to promoting reproductive longevity

Type of Intervention (alphabetical)	Age of Initiation	Dose & Duration	Organism and/or Cell Type	Reproductive Outcome	References
2-deoxyglucose	NA	NA	Human and mouse ovarian cells	Increased FOXO3 activity; reduction in transition from primordial to primary follicles	Barilovits, S. J. *et al.* Characterization of a mechanism to inhibit ovarian follicle activation. *Fertil. Steril.* (2014). doi:10.1016/j.fertnstert.2014.01.025

Coenzyme Q10	36 weeks	22 mg/kg three times weekly for 12 weeks	Mice	Increased primordial follicle count; increased litter size; increased pool of respiring mitochondria; normalized mitochondrial membrane potential; increased ATP output	Ben-Meir, A. *et al.* Coenzyme Q10 restores oocyte mitochondrial function and fertility during reproductive aging. *Aging Cell* (2015). doi:10.1111/acel.12368
	< 35 years	200 mg TID for 60 days	Humans with diminished ovarian reserve	Increased number of oocytes retrieved; improved fertilization rate; improved embryo quality	Xu, Y. *et al.* Pretreatment with coenzyme Q10 improves ovarian response and embryo quality in low-prognosis young women with decreased ovarian reserve: A randomized controlled trial. *Reprod. Biol. Endocrinol.* (2018). doi:10.1186/s12958-018-0343-0
Curcumin	12-16 weeks	100 mg/kg/day for 14 days	Wistar albino rats exposed to cyclophosphamide	Improved ovarian histopathologic appearance after cyclophosphamide and decreased follicular atresia	Melekoglu, R., Ciftci, O., Eraslan, S., Cetin, A. & Basak, N. Beneficial effects of curcumin and capsaicin on cyclophosphamide-induced premature ovarian failure in a rat model. *J. Ovarian Res.* (2018). doi:10.1186/s13048-018-0409-9
	Unspecified	200 mg/kg IP once	Wistar albino rats exposed to ovarian ischemia/reperfusion injury	Increased AMH levels	Eser, A. *et al.* Protective effect of curcumin on ovarian reserve in a rat ischemia model: An experimental study. *Clin. Exp. Obstet. Gynecol.* (2017). doi:10.12891/ceog3235.2017
	7-8 weeks	100 mg/kg/day for 42 days	C57/Bl6 mice exposed to galactose	Decreased FSH and increased estrogen levels; increased AMH mRNA expression; increased	Yan, Z. *et al.* Curcumin exerts a protective effect against premature ovarian failure in mice. *J. Mol. Endocrinol.* (2018). doi:10.1530/JME-17-0214

				number of follicles at multiple developmental stages; decreased ovarian cell apoptosis	
	3 weeks	100 mg/kg/day for 33 weeks	NMRI mice	Increased primordial follicle count; increased AMH levels; decreased FSH and increased estrogen levels; improved oocyte maturation; fertilization, and embryo development	Azami, S. H. *et al.* The antioxidant curcumin postpones ovarian aging in young and middle-aged mice. *Reprod. Fertil. Dev.* (2019). doi:10.1071/RD18472
Dietary restriction	10 weeks	55% of standard rodent chow (24% protein, 4% fat, 4.5% crude fiber) for 10 weeks	Sprague-Dawley rats	Increased primordial follicle count; decreased atretic follicles	Li, L. *et al.* Caloric restriction promotes the reserve of follicle pool in adult female rats by inhibiting the activation of mammalian target of rapamycin signaling. *Reprod. Sci.* (2015). doi:10.1177/1933719114542016
	16 weeks	40% caloric restriction for 46 weeks	C57/BL6 mice	Increased primordial follicle count; decreased atretic follicles; increased litter size after 15.5 months of age; increased pup survival rate	Selesniemi, K., Lee, H. J. & Tilly, J. L. Moderate caloric restriction initiated in rodents during adulthood sustains function of the female reproductive axis into advanced chronological age. *Aging Cell* (2008). doi:10.1111/j.1474-9726.2008.00409.x

	14 weeks	40% caloric restriction for 30 weeks	C57/BL6 mice	Increased number of ovulated oocytes; increased number of mature oocytes; decreaesed oocyte aneuploidy; decreased rate of meiotic spindle anomalies; decreased mitochondrial aggregation	Selesniemi, K., Lee, H. J., Muhlhauser, A. & Tilly, J. L. Prevention of maternal aging-associated oocyte aneuploidy and meiotic spindle defects in mice by dietary and genetic strategies. *Proc. Natl. Acad. Sci. U. S. A.* (2011). doi:10.1073/pnas.1018793108
Everolimus	8 weeks	2.5 mg/kg/day five times per week for 4 weeks	C57/BL6 mice exposed to cyclophosphamide	Increased primordial follicle count; increased AMH levels; increased litter size	Goldman, K. N. *et al.* MTORC1/2 inhibition preserves ovarian function and fertility during genotoxic chemotherapy. *Proc. Natl. Acad. Sci. U. S. A.* (2017). doi:10.1073/pnas.1617233114
Genistein	12 months	160 mg/kg/day for 4 months	Sprague-Dawley rats	Increased number of follicles; decrease in atretic follicles	Chen, Z. G. *et al.* Effects of plant polyphenols on ovarian follicular reserve in aging rats. *Biochem. Cell Biol.* (2010). doi:10.1139/O10-012
Growth hormone	23 days	1 mg/kg/day for 7 days starting 3 days prior to radiation	Sprague-Dawley rats exposed to 3.2 Gy whole-body irradiation	Increased AMH levels; Increased primordial follicle counts; decrease in markers of oxidative stress	Mahran, Y. F. *et al.* Growth hormone ameliorates the radiotherapy-induced ovarian follicular loss in rats: Impact on oxidative stress, apoptosis and IGF-1/IGF-1R Axis. *PLoS One* (2015). doi:10.1371/journal.pone.0140055
Melatonin	8-12 weeks	10 mg/kg/night for 12 months	Kunming mice	Increased primordial follicle count; increased litter size; increased number of follicles retrieved; decreased oocyte	Song, C. *et al.* Melatonin improves age-induced fertility decline and attenuates ovarian mitochondrial oxidative stress in mice. *Sci. Rep.* (2016). doi:10.1038/srep35165

				meiotic spindle abnormalities and chromosome misalignment; increased telomere length; reduced mitochondrial ROS generation; reduced apoptosis	
	10 days	10 ug/mL in water at night until death	Sprague-Dawley rats	Delayed puberty; decreased number of rats with abnormal estrous cycles from 180 to 380 days of age	Meredith, S., Jackson, K., Dudenhoeffer, G., Graham, L. & Epple, J. Long-term supplementation with melatonin delays reproductive senescence in rats, without an effect on number of primordial follicles. *Exp. Gerontol.* (2000). doi:10.1016/S0531-5565(00)00092-9
	10 weeks	100 ug/mL in water for 33 weeks	ICR mice	Increased number of oocytes retrieved after superovulation; increased fertilization and blastocyst formation rate; increased number of primordial follicles	Tamura, H. *et al.* Long-term melatonin treatment delays ovarian aging. *J. Pineal Res.* (2017). doi:10.1111/jpi.12381
	24-45 years	3 mg/day for ~ 40 days	Humans undergoing IVF	Improved oocyte morphology; decreased intrafollicular biomarkers of oxidative stress; increased fertilization rate	Tamura, H. *et al.* Oxidative stress impairs oocyte quality and melatonin protects oocytes from free radical damage and improves fertilization rate. *J. Pineal Res.* (2008). doi:10.1111/j.1600-079X.2007.00524.x

	Variable (reproductive-aged)	3-6 mg/day for 40 days	Humans with unexplained infertility undergoing IVF	Increased intra-follicular antioxidant activity (6 mg); increased number of oocytes retrieved (3 and 6 mg); increased oocyte fertilization rate (3 and 6 mg)	Espino J, Macedo M, Lozano G, et al. Impact of melatonin supplementation in women with unexplained infertility undergoing fertility treatment. *Antioxidants*. 2019. doi:10.3390/antiox8090338
	6 weeks	15 or 30 mg/kg for 18 days starting 3 days prior to chemotherapy	ICR mice exposed to cisplatin	Reduction in cisplatin-induced primordial follicle loss; decreased apoptosis in granulosa cells	Jang H, Lee OH, Lee Y, et al. Melatonin prevents cisplatin-induced primordial follicle loss via suppression of PTEN/AKT/FOXO3a pathway activation in the mouse ovary. *J Pineal Res*. 2016. doi:10.1111/jpi.12316
	Perimenopausal (42-62 years)	3 mg melatonin/day for 6 months	Perimenopausal humans	Decrease in FSH levels; resumption of menstrual cyclicity in 6 postmenopausal women	Bellipanni, G., Bianchi, P., Pierpaoli, W., Bulian, D. & Ilyia, E. Effects of melatonin in perimenopausal and menopausal women: A randomized and placebo controlled study. *Exp. Gerontol.* (2001). doi:10.1016/S0531-5565(00)00217-5
Metformin	28 weeks	100 mg/kg/day for 6 months	C57/BL mice	Increased primordial follicle count; increased proportion of regular estrous cycles	Qin, X. *et al.* Metformin prevents murine ovarian aging. *Aging (Albany. NY).* (2019). doi:10.18632/aging.102016
mTORC1/2 inhibitor INK128	8 weeks	0.3 mg/kg five times per week for 4 weeks	C57/BL6 mice exposed to cyclophosphamide	Increased primordial follicle count; increased litter size	Goldman, K. N. *et al.* MTORC1/2 inhibition preserves ovarian function and fertility during genotoxic chemotherapy. *Proc. Natl. Acad. Sci. U. S. A.* (2017). doi:10.1073/pnas.1617233114

N-acetyl-L-cysteine	NA	0.6 mM concentration added to culture medium	Postovulatory mouse oocytes	Decreased intracellular oocyte ROS; increased intracellular ATP levels; decreased frequency of oocyte meiotic spindle defects; decreased abnormal distribution of cortical granules	Wang, Y. *et al.* N-acetyl-L-cysteine (NAC) delays post-ovulatory oocyte aging in mouse. *Aging (Albany. NY).* (2019). doi:10.18632/aging.101898
	NA	5 and 30 mM concentration added to embryo culture	Mouse embryos exposed to cigarette smoke condensate or cadmium	Improved rate of zygote cleavage; improved rate of survival to blastocyst; reduced apoptosis; decreased ROS; decreased telomere shortening	Huang, J., Okuka, M., McLean, M., Keefe, D. L. & Liu, L. Telomere susceptibility to cigarette smoke-induced oxidative damage and chromosomal instability of mouse embryos in vitro. *Free Radic. Biol. Med.* (2010)
	NA	30 mM concentration in oocyte culture media	CD1 mouse oocytes exposed to aresenite	Reduction in oocyte meiotic spindle abnormalities and chromosome misalignment	Navarro, P. A. A. S., Liu, L., Ferriani, R. A. & Keefe, D. L. Arsenite induces aberrations in meiosis that can be prevented by coadministration of N-acetylcysteine in mice. *Fertil. Steril.* (2006). doi:10.1016/j.fertnstert.2005.08.060
	4 weeks	0.1 mM and 1mM concentration in water	Kunming mice	Increased number of oocytes after superovulation; increased rate of oocyte fertilization and blastocyst formation; increased litter size up to 9-10 months of age; improved	Liu, J. *et al.* Delay in oocyte aging in mice by the antioxidant N-acetyl-l-cysteine (NAC). *Hum. Reprod.* (2012). doi:10.1093/humrep/des019

				oocyte morphology; increased telomere length	
Oocyte cytoplasmic transfer	NA	NA	Humans with poor embryo quality and/or recurrent implantation failure undergoing IVF	Live birth of 27 infants with mtDNA heteroplasmy	Barritt, J. A., Brenner, C. A., Malter, H. E. & Cohen, J. Mitochondria in human offspring derived from ooplasmic transplantation. *Hum. Reprod.* (2001).<br><br>Cohen, J., Scott, R., Schimmel, T., Levron, J. & Willadsen, S. Birth of infant after transfer of anucleate donor oocyte cytoplasm into recipient eggs. Lancet (1997). doi:10.1016/S0140-6736(05)62353-7
Oocyte spindle transfer	NA	NA	Human oocytes	Blastocyst formation; high rate of abnormal fertilization (52%)	Tachibana, M. *et al.* Towards germline gene therapy of inherited mitochondrial diseases. *Nature* (2013). doi:10.1038/nature11647
Rapamycin	8 weeks; 32 weeks	2 mg/kg/day for 2 weeks	CD1 mice	Increased primordial follicle count; improved oocyte morphology; increased oocyte mitochondrial activity; improved fertility in mating studies after 12 months	Dou, X. *et al.* Short-term rapamycin treatment increases ovarian lifespan in young and middle-aged female mice. *Aging Cell* (2017). doi:10.1111/acel.12617
	10 weeks	5 mg/kg QOD for 10 weeks	Sprague-Dawley rats	Increased primordial follicle count; decreased atretic follicles	Zhang, X. mei *et al.* Rapamycin preserves the follicle pool reserve and prolongs the ovarian lifespan of female rats via modulating mTOR activation and sirtuin expression. *Gene* (2013). doi:10.1016/j.gene.2013.03.039

	8 weeks	5 mg/kg/day starting 1 week before chemo until 1 week after completion	BALB/c mice exposed to cyclophosphamide	Increased number of primordial and growing follicles	Zhou, L. *et al.* Rapamycin Prevents cyclophosphamide-induced Over-activation of Primordial Follicle pool through PI3K/Akt/mTOR Signaling Pathway in vivo. *J. Ovarian Res.* (2017). doi:10.1186/s13048-017-0350-3
Resveratrol	6 weeks	7 mg/kg/day for 12 months	C57/BL6 mice	Increased primordial and primary follicle count; increased number of growing and mature follicles; increased oocyte yield after superovulation; reduced rate of oocyte spindle morphology abnormalities and chromosome misalignment; increased litter size at advanced age; increased telomere length and telomerase activity in ovaries	Liu, M. *et al.* Resveratrol protects against age-associated infertility in mice. *Hum. Reprod.* (2013). doi:10.1093/humrep/des437
	12 months	25 mg/kg/day for 4 months	Sprague-Dawley rats	Increased number of follicles; decrease in atretic follicles	Chen, Z. G. *et al.* Effects of plant polyphenols on ovarian follicular reserve in aging rats. *Biochem. Cell Biol.* (2010). doi:10.1139/O10-012
	Variable (reproductive-aged)	800 mg/day for 40 days	Humans with PCOS	Improved oocyte and embryo morphology during IVF cycle	Bahramrezaie, M. et al. Effects of resveratrol on VEGF & HIF1 genes expression in granulosa cells in the angiogenesis pathway and laboratory parameters of polycystic ovary syndrome: a triple-blind randomized clinical

					trial. J. Assist. Reprod. Genet. 2019. doi:10.1007/s10815-019-01461-6
	8 weeks (2 weeks after chemotherapy treatment)	30 mg/kg/every other day for 2 weeks	C57/BL6 mice treated with busulfan and cyclophosphamide	Decrease in FSH levels; increase in number of follicles	Wu et al., resveratrol alleviates chemotherapy-induced oogonial stem cell apoptosis and ovarian aging in mice. Aging. 2019, Vol 11., No 3.
SRT1720	8 weeks	50 mg/kg/day for 6 weeks	Kunming mice	Increased primordial follicle count	Zhou XL, Xu JJ, Ni YH, et al. SIRT1 activator (SRT1720) improves the follicle reserve and prolongs the ovarian lifespan of diet-induced obesity in female mice via activating SIRT1 and suppressing mTOR signaling. *J Ovarian Res*. 2014. doi:10.1186/s13048-014-0097-z
Tamoxifen	Unspecified	1 mg/kg/day for 6 days starting 3 days prior to radiation	Sprague-Dawley rats exposed to 3.2 Gy whole-body irradiation	Increased AMH levels; increased primordial and antral follicle counts; decreased levels of oxidative stress markers; decreased apoptotic markers; preservation of fertility	Mahran, Y. F., El-Demerdash, E., Nada, A. S., Ali, A. A. & Abdel-Naim, A. B. Insights into the protective mechanisms of tamoxifen in radiotherapy-induced ovarian follicular loss: Impact on insulin-like growth factor 1. Endocrinology (2013). doi:10.1210/en.2013-1214
Visfatin	26-31 weeks	0.1 mL of 5, 10, 100, and 500 ng/mL for 1 dose	C57/BL6 mice undergoing superovulation	Increased blastocyst formation rate (100 and 500 ng/mL) in aged mice; increased litter size in aged mice (all doses)	Choi, K. H. *et al.* Administration of visfatin during superovulation improves developmental competency of oocytes and fertility potential in aged female mice. *Fertil. Steril.* (2012). doi:10.1016/j.fertnstert.2012.02.032

	18 months	0.1 mL of 500 ng/mL or 1000 ng/mL every 2 days for 3 doses	C57/BL6 mice	Increased number of zygotes retrieved (500 ng/mL), improved blastocyst formation rate (500 ng/mL); increased pregnancy rate (500 ng/mL) after superovulation	Park, B. *et al.* Role of Visfatin in Restoration of Ovarian Aging and Fertility in the Mouse Aged 18 Months. *Reprod Sci* (2020). Doi: 10.1007/s43032-019-00074-9
Zygote pronucleus transfer	NA	NA	Abnormally fertilized human zygotes	Increased rate of blastocyst formation *in vitro*	Craven, L. *et al.* Pronuclear transfer in human embryos to prevent transmission of mitochondrial DNA disease. *Nature* (2010). doi:10.1038/nature08958
